# Characteristics of Non-Therapeutic Pregabalin Users Detected by a Community Pharmacies Network in a Region of Southern Europe

**DOI:** 10.3390/jcm13195942

**Published:** 2024-10-06

**Authors:** Maria Perelló, Karla Rio-Aige, Pilar Rius, Francisco J. Pérez-Cano, Manel Rabanal

**Affiliations:** 1 Council of the Pharmacist’s Association of Catalonia, 08009 Barcelona, Spain; mperelloc@gmail.com (M.P.); prius@ccfc.cat (P.R.); 2 Physiology Section, Department of Biochemistry and Physiology, Faculty of Pharmacy and Food Science, University of Barcelona, 08028 Barcelona, Spain; rioaigekarla@ub.edu (K.R.-A.); mrabanal@gencat.cat (M.R.); 3 Institute of Research in Nutrition and Food Safety (INSA), 08921 Santa Coloma de Gramenet, Spain; 4 Directorate-General for Healthcare Planning and Regulation, Ministry of Health, Government of Catalonia, 08028 Barcelona, Spain

**Keywords:** pregabalin, prescription drug abuse, misuse, community pharmacy, neuropathic pain

## Abstract

**Background**: Since 2008, several cases of pregabalin abuse have been reported to the European Monitoring Centre for Drugs and Drug Addiction (EMCDDA). Despite this evidence, gabapentinoids are increasingly being prescribed. Moreover, pregabalin is being used in a recreational setting for its dissociative effects and euphoria. **Objectives**: To assess the characteristics of non-therapeutic users of pregabalin and to show behavioral trends associated with requests for the medicine at community pharmacies. **Methods**: The Medicine Abuse Observatory (MAO), an epidemiological surveillance system, was able to analyze trends about the most diverted drugs and the behavioral patterns of the population from community pharmacies. We have conducted an observational and cross-sectional study from January 2022 to April 2023, to determinate trends in the behavior of patients who have requested pregabalin in the Catalan Sephanet. **Results**: Behavior with respect to sex was similar in all health problems, although one difference was raised when considering neuropathic pain, in which the females were more involved (72.7%), especially around 2.5 times more than the males (27.3%, *p* < 0.05). The study showed a potential recreational use related to patients aged <25 years and patients aged 25–35 years (*p* < 0.05). Neuropathic pain was mainly identified in patients >65 years. In 75% of the cases, there was a preceding prescription. **Conclusions**: This study underlines the evidence of non-therapeutic use of pregabalin among the Catalan population and the need to take control measures. Actions should be promoted, both at the level of prescription and dispensing, and focusing on education and knowledge about the risks that may appear with the use of pregabalin.

## 1. Introduction

Pregabalin is a structural derivative of the inhibitory neurotransmitter gamma-aminobutyric acid (GABA). Along with gabapentin, it belongs to the class of gabapentinoids. The activity of gabapentinoids is due to binding with an auxiliary subunit of voltage-dependent calcium channels, in the neurons of the central nervous system, which causes a reduction in the entry of calcium into the nerve terminals, and therefore a decrease in excitability [1].

Gabapentinoids are widely used for the treatment of neuropathic pain and epilepsy. Additionally, pregabalin is also approved for generalized anxiety disorder and for fibromyalgia. Furthermore, gabapentinoids are used off-label for a variety of conditions, including various psychiatric and substance use disorders, increasing their accessibility among vulnerable patients [1,2]. Although both substances share some mechanisms of action, they have some pharmacokinetic differences that could explain the different potential for abuse. For example, pregabalin has a higher absorption rate and bioavailability, suggesting that misuse and abuse occur more frequently compared with gabapentin [2].

Pregabalin treatment can start with a dose of 150 mg per day given as two or three divided doses, and the maximum dose of 600 mg per day may be achieved after an additional seven-day interval. The most common adverse effects are dizziness, somnolence, and a euphoric mood, as a common psychiatric disorder. Evidence from preclinical and therapeutic clinical trials suggests the development of tolerance to the euphoric effects. In addition, pregabalin can enhance the sedative effects of central-nervous-system depressants such as barbiturates, ethanol, and opioids. Special warnings and precautions may occur at therapeutic doses and include suicidal ideation, misuse, abuse, or dependence [1,3].

Pregabalin was approved in the European Union and the US in 2004. Since 2008, several cases of pregabalin abuse were reported to the European Monitoring Centre for Drugs and Drug Addiction (EMCDDA). Most patients with pregabalin use are also currently or have previously been dependent on other substances [3]. However, the warning for an abuse liability was first reported by a study based on pharmacovigilance data in 2010 [4]. Since then, a growing body of evidence has been accumulating [5,6,7]. Data from the European Medicines Agency’s EudraVigilance, between 2004 and 2015, indicated a sharp rise in pregabalin cases of dependence and abuse compared to previous years [8].

Shortly after initial approval, pregabalin was classified as a Schedule V drug by the US Drug Enforcement Agency (DEA) [9]. In 2016, in the UK, the Advisory Council on the Misuse of Drugs (ACMD) raised concerns over its medical misuse, illegal diversion of the drugs, and addiction, and recommended that gabapentin and pregabalin should be controlled as class C drugs under the Misuse of Drugs Act. So the government announced in October 2018 that the prescription drugs pregabalin and gabapentin would be reclassified as class C controlled substances in 2019 [10].

Despite evidence of abuse, gabapentinoids are increasingly being prescribed. The Spanish Agency of Medicines and Medical Devices (AEMPS) report about outpatient use of antiepileptics in the period 2008–2016 highlighted an increase in pregabalin, which accounted for 38.4% of use in its therapeutic group in 2016 [11]. It is suggested that prescribers may be advocating the use of these off-label medicines to avoid prescribing opioid analgesics, resulting in increased diversion [2]. In the UK, gabapentin and pregabalin prescription increased by 350% and 150% over five years, respectively [12]. In Australia gabapentin was ranked sixth in the top financed drugs in 2016–2017 [13].

A systematic review by Evoy et al. showed that misused gabapentinoids are most often obtained from healthcare providers, family, acquaintances, and internet purchase. Recently, an update confirmed that gabapentinoids are increasingly abused or misused to self-medicate [2,6].

Pregabalin is being used in recreational settings for its dissociative effects and euphoria, which seems to be a dose-dependent adverse effect, occurring independently of indication and previous abuse of substances [8,14,15]. Additionally, abuse of higher doses of this drug (up to 20 times higher than the maximal dosage indicated) has been reported. This mostly seems to occur by oral intake. However, intravenous and nasal insufflation have also been observed [3]. The risk of overdose death from pregabalin is low. However, there is a concern about the combination of pregabalin with opioids and benzodiazepines, since opioid users report that pregabalin reinforces the effects of opioids and reduces the undesirable effects of withdrawal symptoms [6,7,12,14,16,17,18,19].

A recent article aimed to determine the prevalence of the prescription of gabapentinoids for nonmedical use. It showed that chronic pain, use of illicit substances, and history of substance abuse treatment were the principal reasons [7]. It also observed that the risk of misuse is higher in psychiatric patients [16,20].

Additionally, a recent study that characterized patterns of pregabalin users from substance abuse treatment facilities detected changes in the users’ profile over the study period. It confirmed that the users increased their precarity, dependence, and use of a higher dose of pregabalin [21]. Similar data were obtained by Servais et al., who highlighted a profile of young male users, immigrants with precarious living conditions and difficulties with their daily situations [22].

In this context, the aim of this study is to detect misuse and abuse of pregabalin and identify trends in the behavior of patients who request the medication at a community pharmacy in Catalonia.

## 2. Materials and Methods

### 2.1. Study Setting

Pharmacists can promote the safe use of medicines, providing medication review services, especially in chronic treatments, as well as carryout epidemiological surveillance activities. In 2017, the Medicine Abuse Observatory (MAO) was set up in Catalonia as a project supported by the Catalonia Pharmacists Council and the Ministry of Health of the Government of Catalonia, to analyze trends about the most diverted drugs and the behavioral patterns of the population from community pharmacies [23]. The 75 pharmacies included constituted a proportional stratification of the population in Catalonia and belonged to the Catalan sentinel pharmacy network (Catalan Sephanet) [24].

The MAO was able to identify changes in behavior with respect to the non-therapeutic use of certain drugs in the health emergency experienced [25]. From there, follow-up studies were carried out on specific medications. In this context, we have conducted an observational and cross-sectional study from January 2022 to April 2023 to determine trends in the behavior of patients who requested pregabalin in the Catalan Sephanet.

### 2.2. Study Design

The MAO performed the approach, which allowed collecting data on the abuse and misuse of medications through a screening tool based on questionnaires. This information became a key source for identifying some behavioral patterns regarding the abuse or misuse of pregabalin. A validated questionnaire was created to identify the signs and behaviors that gave a clearer indication that a drug abuse existed. We based our study on an approach proposed by Finch in 1993, which provided a theoretical basis to link these constructs and the determinants of people who presented drug-seeking behavior [26]. These elements included a pattern of calling for refills after some hours and/or repeatedly requiring early refills, prescriptions from multiple doctors, frequent visits to emergency rooms, strong preference and knowledge for a particular medicine, and incongruence between the severity of the complaint and the physical presentation. The questions consisted of an anonymous multiple-choice test that contained 11 closed and two open-ended questions (Table 1). The issues included in the questionnaire were referred to the identification of the pharmacy (question 1), the demographic profile of the patient, and information related to the health problem and the medicine (questions 2 to 6). The questionnaire also asked for the criteria of suspicion and the medicine request type (questions 7 and 8) if they had previously used it, the treatment duration (questions 9 and 10), and the pharmacist management (question 11). Finally, the two open-ended questions were included in order to discover the reason in case of providing the medicine to the patient, and finally a section of observations was also present.

### 2.3. Data Collection

Specific teaching was carried out by the Pharmacists College of Barcelona (COFB) in order to train pharmacists, to provide information about this context, and to standardize the procedure for data collection. Additionally, throughout the study period, pharmacists received multiple training sessions to reinforce the concepts and resolve any questions. The training program consisted of a 90-min interactive session focused on theoretical frameworks and operational procedures.

The pharmacist filled out the questionnaire only in cases where a customer who requested medicine displayed two or more of the established signs and behavioral symptoms and was suspected of abusing the medicine. Patient information was obtained anonymously by observation. The pharmacist, in some cases, could ask about previous use of the medication or how long it had been taken as part of the patient’s pharmacist interview for pharmacotherapeutic monitoring. As no personal data were collected, neither verbal nor written consent was required.

A survey based in web format and the collection of data software called Typeform V1 (Typeform SL, Barcelona, Spain) consisted in an easy-to-use questionnaire based on closed and open-ended questions. The pharmacist had access to the questionnaire through a link accessible through the COFB website, which is the main online work tool for pharmacists in this area. This software converted the data into an Excel spreadsheet (2021, Microsoft 365), thus enabling further statistical operations and analysis.

### 2.4. Data Analysis

Patient characteristics that were categorical variables were summarized as counts and percentages. The χ^2^ test was used for this purpose, and a *p*-value < 0.05 was considered statistically significant. The analyses were conducted with SPSS software, version 18 (SPSS Inc., Chicago, IL, USA). To find similarities in the individual profile, a multiple correspondence analysis (MCA) was also performed by R version 4.1.2 (R Foundation, Vienna, Austria) (https://www.R-project.org/) using the packages FactoMineR (https://cran.r-project.org/web/packages/FactoMineR/index.html) for the analysis and factoextra for the visualization (https://cran.r-project.org/web/packages/factoextra/index.html). Two categories that present high coordinates and are close in space are directly associated with each other. When the cos2 value for one variable category was close to one, this indicates that it is well represented by two dimensions. On the basis of the MCA results, two profiles were analyzed in greater depth and an odds ratio (OR) with 9 5% confidence interval (CI) was used to calculate the prevalence of different variables.

## 3. Results

### 3.1. Medicinal Product and Patient Profile

Out of the 97 questionnaires received, 80 were included in the study (82.5%) and 17 questionnaires were excluded because they did not consider cases according to the inclusion criteria or because the pharmacist did not provide enough information.

#### 3.1.1. Global Pregabalin User Profile

**Figure 1:**
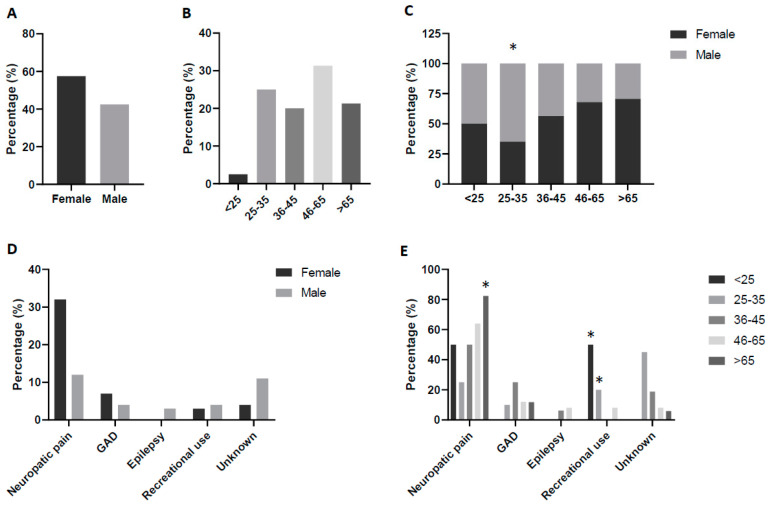
Profile of the pregabalin user. Distribution of participants according to (**A**) sex, (**B**) age, (**C**) age and sex, (**D**) health problem and sex, and (**E**) health problem and age. Values are expressed as percentages for each condition related to 80 notifications. Statistical differences: (**C**) * *p* < 0.05 men vs. women in same age interval or health condition.

The pregabalin users’ distribution profile was not equally distributed in terms of sex and age (Figure 1A,B). Regarding sex, they were mostly women (57.5%) and when age is considered, the highest proportion of users was found in the age interval of 45–65 years (31.3%), followed by 25–35 years (25%), >65 years (21.3%), and 36–45 years (20%). The lowest proportion of prescription drug users was found in the youngest interval of age considered (<25 years, 2.5%). The combination of age and sex data allowed us to better characterize the profile of the patients showing a predominance of male in the age range of 25–35 years (*p* < 0.05 vs. the other groups of age) (Figure 1C).

Considering the health problems for which the pregabalin was requested (Figure 1D), neuropathic pain was notified in 55% of cases, generalized anxiety disorder in 13.8%, potential recreational use in 8.8% and epilepsy in 3.7%. In 18.7% of cases the pharmacist could not identify the motive of the pregabalin request. Behavior in terms of sex was similar in all the health problems, and only one difference raised when considering neuropathic pain, in which the females were more involved (72.7%), specifically around 2.5 times more than the males (27.3%, *p* < 0.05). In addition, the 100% reported cases of epilepsy implicated men (Figure 1D).

The combination of health problems stratified by age showed a potential recreational use related to patients aged <25 years and 25–35 years (*p* < 0.05 vs. the other groups of age). On the other hand, neuropathic pain was mainly identified in patients >65 years (*p* < 0.05 vs. the other age groups) (Figure 1E).

#### 3.1.2. Pregabalin Requested Approach

**Figure 2:**
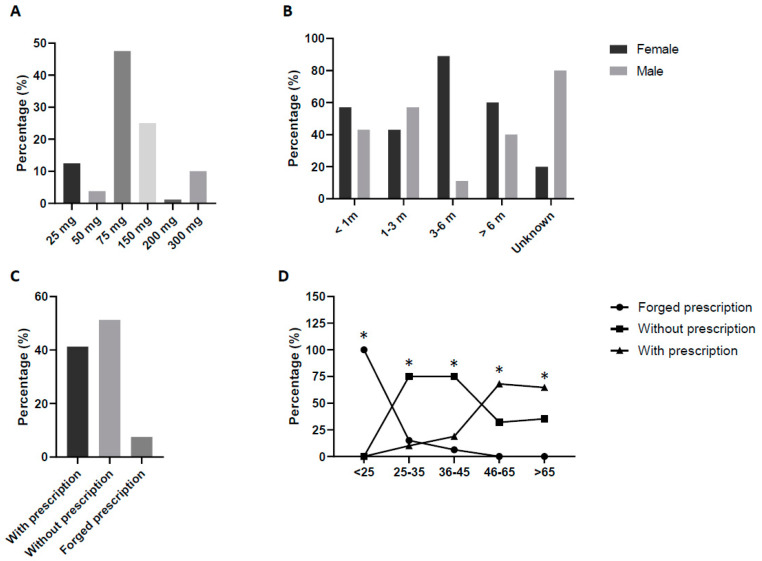
Approach to pregabalin requests. Distribution of pregabalin users regarding (**A**) dose, (**B**) treatment duration, (**C**) drug request, and (**D**) medicine request type and age. Proportions are calculated from each condition. Statistical differences: (**D**) * *p* < 0.05 for same age interval in different condition.

The number of reported cases of potential drug abuse (Figure 2A) was the highest for the 75 mg dose (47.5%) followed by 150 mg (25%), 25 mg (12.5%), and 300 mg (10%). The lowest values were found in the 50 mg (3.75%) and 200 mg (1.25%) requests. In terms of health problem and medicine dose, no differences were observed except for users identified for potential recreational use that were linked to the 150 mg dose compared to all other doses (*p* < 0.05). With respect to age, the 75 mg dose was associated with those over 65 years of age (*p* < 0.05). The main drug involved was Lyrica^®^ (76.3%). Another relevant piece of data obtained was the duration that the patients had been taking the drug. Most had been more than 6 months (56.25%), followed by 1–3 months (17.5%), 3–6 months (11.25%), and <1 month (8.75%) (Figure 2B). No differences were detected regarding age and sex, with exception of the 3–6 months of duration that were more linked to women (89%) than men (11%) (*p* < 0.05).

The type of drug request was also considered (Figure 2C). Drugs were most requested without prescription (51.25%), followed by a formal prescription (41.25%). Moreover, in 7.5% of the answers, the drug was requested with a probable forged prescription.

Although no differences were detected regarding sex or requested dose, these proportions were not the same among all the age groups studied, and patients aged <25 were associated with falsified prescriptions in all cases (*p* < 0.05 vs. the other groups of age). In contrast, patients >65 years of age showed a significant association with formal prescription demands compared to the other age groups (*p* < 0.05). In addition, those aged 25–35 requested the medicine without a prescription in more cases than the other age groups (*p* < 0.05) (Figure 2).

#### 3.1.3. Reported Suspicious Criteria and Pharmacist Management

Another important variable considered was whether requests were frequent or not. Overall, an approximately 50/50 proportion was found for frequent/nonfrequent requests. The proportion of frequent requests was significantly higher in patients aged 46–65 than in other ages (*p* < 0.05), without differences regarding the dose.

**Figure 3:**
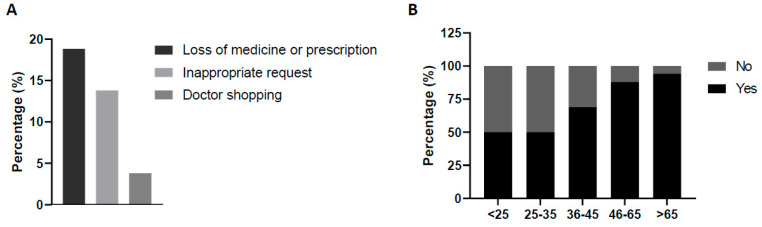
Proportion of the reported suspicious criteria. Distribution related to (**A**) user behavior or (**B**) previous use. Statistical differences: (**B**) *p* < 0.05 for the age interval of 25–35 years and absence of previous use vs. the other age interval groups.

Other suspicion criteria were identified in 36.3% of cases. The loss of medicine or prescription to try to get the pregabalin accounted for 18.75% of cases and was linked to women (85.7%) (*p* < 0.05). Also, nearly 14% reported an inappropriate demand. The experience of doctor shopping was uncommon (Figure 3A). In relation to age and claim, no differences were observed between groups, and only the patients aged 25–35 years showed a higher trend of inappropriate requests compared to the other groups (25%) (*p* = 0.09).

Regarding the previous use of the substance, in 75% of the cases there was a preceding prescription. However, if we consider age, patients <25 years old and those 25–35 years old stated in 50% of cases that they did not have a previous prescription for the drug (*p* < 0.05) (Figure 3B).

The pharmacist only provided the medicinal product to the patient in 18.75% of the cases. The qualitative analysis carried out of the open-ended question of the questionnaire (“Why do you supply the medicine?”) showed that the main reasons for doing so reported by pharmacists were that patients struggled to access general practice or were well-known patients.

### 3.2. Overall Pregabalin User Characteristics

**Figure 4:**
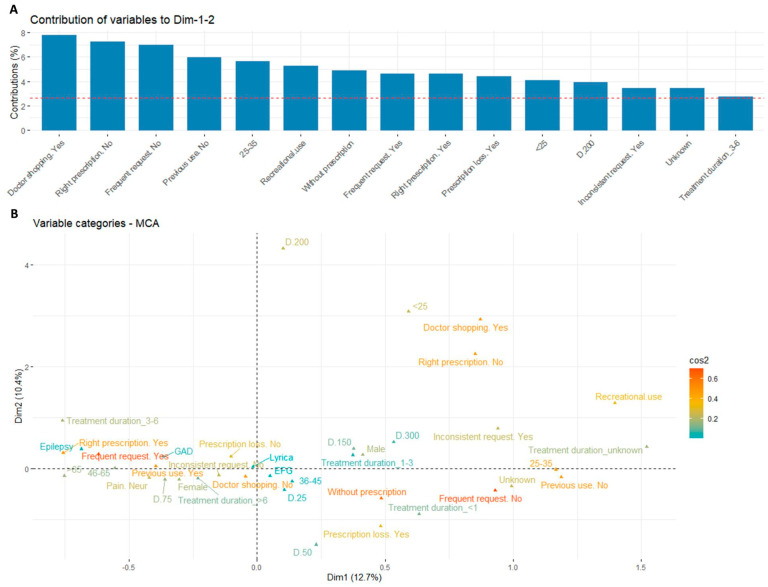
Multiparametric analysis of all variables analyzed regarding pregabalin notifications. Variables are questions/responses pairs from the information collected using the questionnaire from Table 1. Percentage of explained variances for each dimension in the model (**A**). Association between the variables in the first two dimensions (**B**). Values are derived from the dataset of 80 participants.

All the validated notifications of the present study allowed us to obtain information about the patient profile and behavior and the characteristics of the medicinal product. The participants’ features were multi-parametrically approached to analyze similar profiles between the individuals in the study and to evaluate associations between variable categories by multiple correspondence analysis (MCA) (Figure 4A). The model provided a variance of 12.7% for first and 10.4% for second dimensions (Dim-1 and Dim-2, respectively) (Figure 4A).

The contribution of each variable in Dim1 and Dim2 was analyzed, allowing us to observe that “Age” and “Frequent request” were the most correlated with Dim-1 and “Request type” with Dim-2 (Figure 4A). This finding was in line with the visualization of the results in Figure 4B where it can be observed that some frequent demand and type request categories had the highest COS2 value, such as “Right prescription. No”.

### 3.3. Analysis Based on the Frequency of Medication Requests

On the basis of the MCA results, the patient and medicinal product profiles were analyzed in greater depth based on whether the medicinal product was requested with frequency or not (Table 2).

The patient distribution profile was equally distributed between the sexes. Regarding the age of the patients, being aged between 45 and 65 is related to a frequent request for pregabalin (OR: 5.44, 95% CI: 1.65–17.99). By contrast, the age range of 25–35 was only identified in 20% of cases. Another important variable was health problem. No differences were observed between frequent and nonfrequent users about the proportion of cases of neuropathic pain, epilepsy, generalized anxiety disorder, and potential recreational use, and, in both the cases of asked with frequency or not, neuropathic pain was the most involved problem.

Concerning the medicinal product form and dose, those of the Lyrica^®^ brand and 75 mg were most reported in both high frequency requests or not, without a differential pattern. A statistically significant difference was observed concerning how the medicinal product was requested. In this way, users who frequently requested pregabalin brought a prescription in most cases, unlike punctual users who asked for it without prescription (OR: 17.33, 95% CI: 4.50–66.62).

Regarding the suspicion criteria presented by the patient when requesting the substance, an inappropriate request and the loss of the prescription or the medicine were observed in greater rate in those punctual requests (OR: 5, 95% CI: 1.21–20.62 and OR: 32.16, 95% CI: 3.92–263.22), respectively.

In addition, a higher previous use of the drug was identified in requests made frequently compared to nonfrequent ones (OR: 7.59, 95% CI: 2.39–24.22).

In relation to treatment duration, as expected, it was observed that high-frequency users were associated with longer treatment durations (3–6 months and >6 months) whereas punctual users reported shorter treatments (<3 months). The management and attitude of pharmacists when addressing these requests was similar in both situations, and in ∼80% of cases the dispensation did not occur.

### 3.4. Analysis Based on the Type Request

The patient and medicinal product profile analysis was also performed based on whether the medicinal product was requested with a prescription or not (Table 3). In general, non-prescription requests included a 25- to 35-year-old profile associated with the male sex and doses of 150 mg (*p* < 0.05 vs. the other age groups, females or doses).

The patient distribution profile was similar regarding sex, but different related to age. A higher proportion of people aged 46–65 years asked for the substance with a prescription (51.5%, *p* < 0.05 vs. the other age groups). On the other hand, a higher proportion of younger people (25–35 years and 36–45 years) requested the drug without prescription (36.6% and 29.3%, respectively, *p* < 0.05 vs. the other age groups). A statistically significant difference was observed regarding health problem. Patients who reported neuropathic pain (N = 44, 55%) showed a significant trend to use a prescription to request pregabalin (OR: 2.66, 95% CI: 1.02–6.97).

Moreover, the proportion of frequent request was different between those that did not provide a prescription and those that have it, as was detailed above. With regard to previous use, it was a condition linked to those who requested the pregabalin with prescription (OR: 6.4, 95% CI: 1.67–24.51). Other variables as an inappropriate demand, specific doses, or treatment duration were similar between both situations, with the exception of treatments of less than one month that were only reported in requests without prescription.

## 4. Discussion

The main finding of this study is that it confirms the phenomenon of misuse and abuse of pregabalin among the population in this southern region of Europe. The results show that neuropathic pain followed by generalized anxiety disorder was the main reason reported for nonmedical use. This could indicate that misuse was associated with self-medication and even withdrawal from other medications [2,27].

Although the published data uphold the opinion that the role of age, gender, and socioeconomic status in pregabalin misuse remains unclear [28], this study highlighted neuropathic pain condition and its association with women and patients over 65 years old. Additionally, the data showed that users between 25 and 35 years of age tended to be male and to engage in recreational use of pregabalin. The latter was also found in users under 25 years of age. This is in line with some data, which describe that young men are the most likely abusers of pregabalin, sometimes associated with other substances [14,16,29]. Indeed, in accordance to some evidence, opioids, benzodiazepines, and illicit drugs were commonly taken together in those who overdosed on pregabalin [30].

Regarding the dose, a higher dose of 150 mg was associated with recreational use, which could indicate that the use of supratherapeutic doses is used to achieve euphoria [2] but which also may reflect that the doctor prescribed daily doses higher than recommended because of insufficient pain relief [31]. The way in which the drug is requested, with or without a prescription or through a forged prescription, may be an indicator that the patient is an abuser. Thus, the information provided by this study allowed us to observe different associated patterns: Patients under 25 requested the medicine through a forged prescription, patients between 25 and 35 without a prescription, and those over 65 with a prescription. In this case, we observed an association with the prescribed dose of 75 mg. In general, however, the study has not enabled us to conclude whether the prescribed dose is a determinant factor for misuse or abuse, probably because the number of subjects analyzed was not sufficient when they are stratified by dose. These data are similar to those presented by a French study showing almost half of the cases of pregabalin being requested by a valid prescription, which supports the growing ease of access to this substance [16].

Another area considered was duration of treatment. In more than half of the cases, the treatment was greater than 6 months, which could be linked to the chronicity of the pain or other conditions. This agrees with a study by the Eudravigilance database which outlined that there was an increase in global reporting of pregabalin over time, especially abuses and dependencies [8]. When analyzing the suspicious criteria, it was highlighted that pharmacists in several cases had reported the loss of medicine or prescription or an inappropriate request. This last condition was especially found for those between the ages of 25 and 35, which is in line with other studies suggesting deviant behaviour as an important part of this misuse, such as the use of forged prescriptions [16,31]. In addition, frequency of demand, which was associated with the previous use of pregabalin, might suggest the existence of tolerance.

Actually, this study also highlights that patients aged 46–65 years are associated with frequency of request and treatments of more than 6 months, which may be a risk factor for the abuse of gabapentinoids [29,32]. Known data about lifetime prevalence of gabapentinoid misuse in the general population described 1.1% for gabapentin and 0.5% for pregabalin [2]. Also, a US national survey estimated that 6.6% of people had misused a gabapentinoid during their lifetime [15,33].

Within this context, a report found that the rising number of pregabalin exposures correlated with the rising prescription rate, which is occurring in developed countries across the world [34]. In addition, a systematic review suggested that rapid dose titration schedules, low cost, misunderstanding of abuse potential among prescribers, and frequent off-label use increase the number of patients exposed to the drug [2]. In fact, according to Pharma Marketing (2018), worldwide sales of pregabalin (Lyrica) in 2017 reached 10th position in terms of gross sales, with an annual growth rate of about 2.8% [3]. Moreover, referring to seizures carried out in the European Union, in 2021 pregabalin accounted for 3% (235 kg) of the new psychoactive substances [35]. The increased use of pregabalin in Europe calls for greater vigilance in the general population and specifically among patients with substance use disorders or patients with chronic pain [31]. In this line, in Spain, gabapentinoid use continues to rise and the latest report, with data up to 2022, informs us that the percentage of DHD (daily dose defined per 1000 inhabitants per day) of pregabalin goes from 69.3% in 2019 to 70.6% in 2022, and even exceeds metamizole [36].

Overall, this study underlines the evidence of non-therapeutic use among the Catalan population and the need to take control measures, given that the increase in its use has been noticed throughout the last few years, either because of the increase in prescription (possibly due to off-label indications) or because of its recreational use, as referred by some studies and media [7,37].

The present study has several limitations. On the one hand, the small sample size may not accurately identify significant relationships in the data. Despite this, it allows us to describe the trends and the behavior associated with pregabalin abuse and misuse regarding patients who request the medicine at the community pharmacy. On the other hand, and even more critical, the design of the study does not include clinical data regarding the patients. Thus, the results cannot allow us to infer causality regarding drug choice and other variables and conditions such as comorbidity, polytherapy, or some health situations or living conditions, although it provides a patient profile on gender and age.

## 5. Conclusions

This study performed from pharmacies has evidenced the phenomenon of the non-therapeutic use of pregabalin, the users’ characteristics, and the behavior associated with this abuse and misuse. Thus, surveillance from community pharmacies can be a key element to provide this type of information and to share with other health professionals. Actions should be promoted, both at the level of prescription and dispensing, and focusing on education and knowledge about the risks and possible side effects that may appear with the use of pregabalin.

Further research is needed to better understand the impact of environmental factors such as comorbidities, polytherapy, and living conditions on this phenomenon.

## Figures and Tables

**Table 1:** Questionnaire to evaluate potential pregabalin abuse and misuse.

Question Number	Question Text	Answer
1	Pharmacy ID	
2	Patient sex	Male/Female
3	Patient age	<25/25–35/36–45/46–65/>65
4	Medicinal product form	Lyrica^®^/Generic drug/Others
5	Medicinal product dose	25 mg/50 mg/75 mg/150 mg/200 mg/300 mg
6	Health problem	Neuropatic pain/Epilepsy/Generalized anxiety disorder/Recreative use/Don’t know
7	Suspicion criteria	Frequent demand/Doctor shopping/Inappropriate request/Loss of prescription or medicine/Others
8	Drug request	Requested with prescription/Requested without prescription/Probably forged prescription
9	Previous use	Yes/No
10	Treatment duration	<1 month/1–3 month/3–6 month/>6 month
11	Pharmacist management	Supplied/Not supplied
12	Why do you supply the medicine?	
13	Observations	

**Table 2:** Analysis of the patient and medicinal product profiles based on whether or not the medicinal product was requested with frequency. Values are expressed as percentages for each condition in a total of 80 participants.

Variable	Item	Punctual Users N = 32	High Frequent Users N = 48
Sex	Female	18 (56.3%)	28 (58.3%)
	Male	14 (43.7%)	20 (41.7%)
Age	<25	0 (0%)	2 (4.2%)
	25–35	16 (50%)	4 (8.3%) *
	36–45	8 (25%)	8 (16.7%)
	46–65	4 (12.5%)	21 (43.7%) *
	>65	4 (12.5%)	13 (27.1%)
Health problem	Neuropathic pain	14 (43.7%)	30 (62.5%)
	Epilepsy	0 (0%)	3 (6.2%)
	Generalized anxiety disorder	3 (9.4%)	8 (16.7%)
	Recreative use	5 (15.6%)	2 (4.2%)
	N/A	10 (31.3%)	5 (10.4%)
Medicinal product	Lyrica	22 (68.8%)	39 (81.3%)
	Pregabalin EFG	10 (31.2%)	9 (18.7%)
Medicinal product dose	25 mg	5 (15.6%)	5 (10.4%)
	50 mg	3 (9.4%)	0 (0%) *
	75 mg	12 (37.5%)	26 (54.2%)
	150 mg	7 (21.9%)	13 (27%)
	200 mg	0 (0%)	1 (2.1%)
	300 mg	5 (15.6%)	3 (6.3%)
Drug request	With prescription	3 (9.4%)	30 (62.5%) *
	Without prescription	26 (81.2%)	15 (31.2%) *
	Probably forged prescription	3 (9.4%)	3 (6.3%)
Doctor shopping	Yes	2 (6.3%)	2 (4.2%)
	No	30 (93.7%)	46 (95.8%)
Inaproppriate request	Yes	8 (25%)	3 (6.3%) *
	No	24 (75%)	45 (93.7%) *
Loss of prescription or medicine	Yes	13 (40.6%)	1 (2.1%) *
	No	19 (59.4%)	47 (97.9%) *
Treatment duration	<1 m	5 (15.6%)	2 (4.2%)
	1–3 m	8 (25%)	6 (12.5%)
	3–6 m	0 (0%)	9 (18.7%) *
	>6 m	15 (46.9%)	30 (62.5%)
	N/A	4 (12.5%)	1 (2.1%)
Previous use	Yes	17 (53.1%)	43 (89.6%) *
	No	15 (46.9%)	5 (10.4%) *
Pharmacist management	Supplied	4 (12.5%)	11 (22.9%)
	Not supplied	28 (87.5%)	37 (77.1%)

* *p* < 0.05 (χ^2^ test) for High Frequent Users group vs. Punctual Users group.

**Table 3:** Analysis of the patient and medicinal product profiles based on whether or not the medicinal product was requested with a prescription. Values are expressed as percentages for each condition in a total of 80 participants.

Variable	Item	Prescription N = 33	No Prescription N = 41
Sex	Female	18 (54.5%)	25 (61%)
	Male	15 (45.5%)	16 (39%)
Age	<25	0 (0%)	0 (0%)
	25–35	2 (6.1%)	15 (36.6%) *
	36–45	3 (9.1%)	12 (29.3%) *
	46–65	17 (51.5%)	8 (19.5%) *
	>65	11 (33.3%)	6 (14.6%)
Health problem	Neuropathic pain	23 (69.7%)	19 (46.3%) *
	Epilepsy	2 (6.1%)	1 (2.4%)
	Generalized anxiety disorder	5 (15.2%)	5 (12.3%)
	Recreative use	2 (6.1%)	3 (7.3%)
	N/A	1 (3%)	13 (31.7%) *
Medicinal product	Lyrica	25 (75.8%)	31 (75.6%)
	Pregabalin EFG	8 (24.2%)	10 (24.4%)
Medicinal product dose	25 mg	3 (9.1%)	6 (14.6%)
	50 mg	0 (0%)	3 (7.3%)
	75 mg	18 (54.6%)	19 (46.3%)
	150 mg	8 (24.2%)	9 (22%)
	200 mg	0 (0%)	0 (0%)
	300 mg	4 (12.1%)	4 (9.8%)
Frequent request	Yes	30 (90.9%)	15 (36.6%) *
	No	3 (9.1%)	26 (63.4%) *
Doctor shopping	Yes	2 (6.1%)	0 (0%)
	No	31 (93.9%)	41 (100%)
Inappropriate request	Yes	2 (6.1%)	6 (14.6%)
	No	31 (93.9%)	35 (85.4%)
Loss of prescription or medicine	Yes	0 (0%)	14 (34.1%)
	No	33 (100%)	27 (65.9%)
Treatment duration	<1 m	0 (0%)	6 (14.6%) *
	1–3 m	6 (18.2%)	6 (14.6%)
	3–6 m	5 (15.1%)	2 (4.9%)
	>6 m	20 (60.6%)	24 (58.6%)
	N/A	2 (6.1%)	3 (7.3%)
Previous use	Yes	30 (90.9%)	25 (61%) *
	No	3 (9.1%)	16 (39%) *
Pharmacist management	Supplied	9 (27.3%)	5 (12.2%)
	Not supplied	24 (72.7%)	36 (87.8%)

* *p* < 0.05 (χ^2^ test) for Non-Prescription group vs. Prescription group.

## Data Availability

The datasets that support the findings of this study are available from the first author (M.P.) upon reasonable written request.

## References

[1] European Medicines Agency (2023). Lyrica, INN-Pregabalin. Summary of Product Characteristics. https://www.ema.europa.eu/en/documents/product-information/lyrica-epar-product-information_en.pdf.

[2] Evoy K.E., Morrison M.D., Saklad S.R. (2017). Abuse and Misuse of Pregabalin and Gabapentin. Drugs.

[3] Expert Committee on Drug Dependence (2018). Critical Review Report: Pregabalin. Geneva: World Health Organization. https://ecddrepository.org/en/pregabalin.

[4] Schwan S., Sundström A., Stjernberg E., Hallberg E., Hallberg P. (2010). A signal for an abuse liability for pregabalin--results from the Swedish spontaneous adverse drug reaction reporting system. Eur. J. Clin. Pharmacol..

[5] Gahr M., Franke B., Freudenmann R.W., Kölle M.A., Schönfeldt-Lecuona C. (2013). Concerns about pregabalin: Further experience with its potential of causing addictive behaviors. J. Addict. Med..

[6] Evoy K.E., Sadrameli S., Contreras J., Covvey J.R., Peckham A.M., Morrison M.D. (2021). Abuse and Misuse of Pregabalin and Gabapentin: A Systematic Review Update. Drugs.

[7] Fonseca F., Lenahan W., Dart R.C., Papaseit E., Dargan P.I., Wood D.M., Guareschi M., Maremmani I., Auriacombe M., Farré M. (2021). Non-medical Use of Prescription Gabapentinoids (Gabapentin and Pregabalin) in Five European Countries. Front. Psychiatry.

[8] Chiappini S., Schifano F. (2016). A decade of gabapentinoid misuse: An analysis of the European Medicine Agency’s suspected adverse drug reactions database. CNS Drugs.

[9] (2005). Drug Enforcement Administration, Department of Justice. Schedules of controlled substances: Placement of pregabalin into schedule V. Final rule. Fed. Regist.

[10] Mayor S. (2018). Pregabalin and gabapentin become controlled drugs to cut deaths from misuse. BMJ.

[11] Agencia Española de Medicamentos y Productos Sanitarios, AEMPS (2017). Utilización de Medicamentos Antiepilépticos en España Durante el Periodo 2008–2016.

[12] Morrison E.E., Sandilands E.A., Webb D.J. (2017). Gabapentin and pregabalin: Do the benefits outweigh the harms?. J. R. Coll. Physicians Edinb..

[13] Cairns R., Schaffer A.L., Ryan N., Pearson S.A., Buckley N.A. (2019). Rising pregabalin use and misuse in Australia: Trends in utilisation and intentional poisonings. Addiction.

[14] Dufayet L., Care W., Deheul S., Laborde-Casterot H., Nisse P., Langrand J., Vodovar D., French PCC Research Group (2021). Increase in pregabalin recreational use in adolescents in France. Clin. Toxicol..

[15] Schjerning O., Rosenzweig M., Pottegård A., Damkier P., Nielsen J. (2016). Abuse Potential of Pregabalin: A Systematic Review. CNS Drugs.

[16] Tambon M., Ponté C., Jouanjus E., Fouilhé N., Micallef J., Lapeyre-Mestre M., French Addictovigilance Network (FAN) (2021). Gabapentinoid Abuse in France: Evidence on Health. Consequences and New Points of Vigilance. Front. Psychiatry.

[17] McAnally H., Bonnet U., Kaye A.D. (2020). Gabapentinoid Benefit and Risk Stratification: Mechanisms Over Myth. Pain Ther..

[18] Hägg S., Jönsson A.K., Ahlner J. (2020). Current Evidence on Abuse and Misuse of Gabapentinoids. Drug Saf..

[19] Gittins R., Vaziri R., Maidment I. (2022). Surveying Over the Counter and Prescription Only Medication Misuse in Treatment Services During COVID-19. Subst. Abus. Res. Treat..

[20] Galliot G., Ponté C., Schmitt L., Hakimi Y., Sergent S., Lapeyre-Mestre M., Salles J. (2022). Case Report: The Comorbidity of Pregabalin-Use Disorder and Post-Traumatic Stress Disorder: Clinical and Pharmacological Issues. Int. J. Ment. Health Addict..

[21] Garnier C., Schein M., Lacroix C., Jouve E., Soeiro T., Gentile G., Lapeyre Mestre M., Micallef J. (2024). Patterns of Pregabalin Users from Substance Abuse Treatment Facilities: Results from the French OPPIDUM Program from 2008 to 2022. CNS Drugs.

[22] Servais L., Huberland V., Richelle L. (2023). Misuse of Pregabalin: A qualitative study from a patient’s perspective. BMC Public Health.

[23] Perelló M., Rio-Aige K., Guayta-Escolies R., Gascón P., Rius P., Jambrina A.M., Bagaria G., Armelles M., Pérez-Cano F.J., Rabanal M. (2021). Evaluation of Medicine Abuse Trends in Community Pharmacies: The Medicine Abuse Observatory (MAO) in a Region of Southern Europe. Int. J. Environ. Res. Public Health.

[24] Jambrina A.M., Rams N., Rius P., Perelló M., Gironès M., Pareja C., Pérez-Cano F.J., Franch À., Rabanal M. (2022). Creation and implementation of a new sentinel surveillance model in pharmacy offices in Southern Europe. Int. J. Environ. Res. Public Health.

[25] Perelló M., Rio-Aige K., Rius P., Bagaría G., Jambrina A.M., Gironès M., Pérez-Cano F.J., Rabanal M. (2023). Changes in prescription drug abuse during the COVID-19 pandemic evidenced in the Catalan pharmacies. Front. Public Health.

[26] Finch J. (1993). Prescription drug abuse. Prim. Care.

[27] Al-Husseini A., Wazaify M., Van Hout M.C. (2018). Pregabalin Misuse and Abuse in Jordan: A Qualitative Study of User Experiences. Int. J. Ment. Health Addict..

[28] Ibiloye E.A., Barner J.C., Lawson K.A., Rascati K.L., Evoy K.E., Peckham A.M. (2021). Prevalence of and Factors Associated with Gabapentinoid Use and Misuse Among Texas Medicaid Recipients. Clin. Drug Investig..

[29] Alshahrani S.M., Orayj K., Alqahtani A.M., Algahtany M.A. (2021). Community Pharmacists’ Perceptions towards the Misuse and Abuse of Pregabalin: A Cross-Sectional Study from Aseer Region, Saudi Arabia. Healthcare.

[30] Mathieson S., Lin C.C., Underwood M., Eldabe S. (2020). Pregabalin and gabapentin for pain. BMJ.

[31] Driot D., Jouanjus E., Oustric S., Dupouy J., Lapeyre-Mestre M. (2019). Patterns of gabapentin and pregabalin use and misuse: Results of a population-based cohort study in France. Br. J. Clin. Pharmacol..

[32] Kiliç Z., Aydin Özaslan E. (2023). Abuse and addiction in gabapentinoid drug users for neuropathic pain. Eur. Rev. Med. Pharmacol. Sci..

[33] Covvey J.R., Blakely M.L., Singh R., Peckham A.M., Evoy K.E. (2023). Pharmacist, prescriber, and drug policy expert opinions on gabapentinoid misuse. Res. Soc. Adm. Pharm..

[34] Isoardi K.Z., Polkinghorne G., Harris K., Isbister G.K. (2020). Pregabalin poisoning and rising recreational use: A retrospective observational series. Br. J. Clin. Pharmacol..

[35] European Monitoring Centre for Drugs and Drug Addiction (2023). European Drug Report 2023: Trends and Developments. https://www.emcdda.europa.eu/publications/european-drug-report/2023_en.

[36] Agencia Española de Medicamentos y Productos Sanitarios, AEMPS (2024). Utilización de Medicamentos Analgésicos no Opioides en España. https://www.aemps.gob.es/medicamentos-de-uso-humano/observatorio-de-uso-de-medicamentos/informes/?lang=ca.

[37] Ortiz-Climent R., Pol-Yanguas E. (2024). Evaluación del uso de gabapentinoides en el contexto del estado español: Etiología del uso y abuso. Bol. Farm. Prescr. Farm. Y Util..

